# Peroxiredoxin-6 Negatively Regulates Bactericidal Activity and NF-κB Activity by Interrupting TRAF6-ECSIT Complex

**DOI:** 10.3389/fcimb.2017.00094

**Published:** 2017-03-24

**Authors:** Yoon Min, Sae M. Wi, Dongwoo Shin, Eunyoung Chun, Ki-Young Lee

**Affiliations:** ^1^Department of Molecular Cell Biology and Samsung Biomedical Research Institute, Sungkyunkwan University School of MedicineSuwon, South Korea; ^2^Department of Immunology and Infectious Diseases, Department of Medicine, Harvard School of Public Health, Harvard Medical SchoolBoston, MA, USA

**Keywords:** peroxiredoxin-6, mROS, bactericidal activity, NF-κB, TRAF6, ECSIT

## Abstract

A TRAF6-ECSIT complex is crucial for the generation of mitochondrial reactive oxygen species (mROS) and nuclear factor-kappa B (NF-κB) activation induced by Toll-like receptor 4 (TLR4). Peroxiredoxin-6 (Prdx6) as a member of the peroxiredoxin family of antioxidant enzymes is involved in antioxidant protection and cell signaling. Here, we report on a regulatory role of Prdx6 in mROS production and NF-κB activation by TLR4. Prdx6 was translocated into the mitochondria by TLR4 stimulation and Prdx6-knockdown (Prdx6^KD^) THP-1 cells had increased level of mitochondrial reactive oxygen species levels and were resistant to *Salmonella typhimurium* infection. Biochemical studies revealed Prdx6 interaction with the C-terminal TRAF-C domain of TRAF6, which drove translocation into the mitochondria. Interestingly, Prdx6 competitively interacted with ECSIT to TRAF6 through its C-terminal TRAF-C domain, leading to the interruption of TRAF6-ECSIT interaction. The inhibitory effect was critically implicated in the activation of NF-κB induced by TLR4. Overexpression of Prdx6 led to the inhibition of NF-κB induced by TLR4, whereas Prdx6^KD^ THP-1 cells displayed enhanced production of pro-inflammatory cytokines including interleukin-6 and -1β, and the up-regulation of NF-κB-dependent genes induced by TLR4 stimulation. Taken together, the data demonstrate that Prdx6 interrupts the formation of TRAF6-ECSIT complex induced by TLR4 stimulation, leading to suppression of bactericidal activity because of inhibited mROS production in mitochondria and the inhibition of NF-κB activation in the cytoplasm.

## Introduction

Reactive oxygen species (ROS) are a component of the killing response to microbial invasion and a mediator of cell signal transduction (Lambeth, 2004; Vogel et al., 2007; West et al., 2011). Phagocytes including macrophages and dendritic cells are specialized cells of the innate immune system, which are designed to engulf and destroy harmful microorganisms in phagosomes (Beutler et al., 2006; West et al., 2006, 2011). Phagocytes produce large quantities of ROS primarily via the phagosomal nicotinamide adenine dinucleotide phosphate (NADPH) oxidase machinery (Lambeth, 2004; Vogel et al., 2007). In addition, the mitochondrial oxidative phosphorylation machinery also contributes to macrophage bactericidal activity through the production of mitochondrial ROS (mROS) (Murphy, 2009; Koopman et al., 2010; West et al., 2011; Geng et al., 2015). The molecular and cellular mechanism by which mROS production is regulated in the mitochondria and is functionally associated with innate immune signaling remain unclear. Two recent reports showed that signals of Toll-like receptors (TLRs) are critical for effective phagosome-mitochondrion function and bactericidal activity (West et al., 2011; Geng et al., 2015). The data revealed that engagement of TLRs in macrophages led to the translocation of mitochondria to phagosomes, which was mediated by the assembly of a complex of the ubiquitin ligase tumor necrosis factor receptor-associated factor 6 (TRAF6) and the mitochondrial complex I–evolutionarily conserved signaling intermediate in Toll pathways (ECSIT), which augmented mROS production and bactericidal activity. In addition, the Mst1 and Mst2 kinases were demonstrated to positively regulate phagocytic induction of ROS and bactericidal activity by promoting TLR-triggered assembly of TRAF6-ECSIT complex (Geng et al., 2015). These results strongly suggest that the functional assembly of TRAF6-ECSIT complex in the mitochondria plays a pivotal role for the production of mROS, and thereby leads to the bactericidal activity.

Beyond the functional role of TRAF6-ECSIT complex in the mitochondria, the complex has also been critically implicated in the activation of nuclear factor-kappa B (NF-κB) induced by TLR stimulation (Kopp et al., 1999; Wi et al., 2014; Mi Wi et al., 2015). ECSIT has been identified and functionally characterized as a novel intermediate in the Toll/Interleukin (IL)-1 signal transduction pathways that bridge TRAF6 to mitogen-activated protein kinase/extracellular signal-regulated kinasekinase-1 (MEKK-1) (Kopp et al., 1999). The adapter ECSIT protein regulates the processing of MEKK-1, which leads to the activation of NF-κB induced by the Toll/IL-1 pathways. Our recent studies have suggested a new regulatory role of ECSIT in the TLR4-mediated signal for the activation of NF-κB (Wi et al., 2014; Mi Wi et al., 2015). In this scenario, ECSIT forms a TAK1-ECSIT-TRAF6 complex induced by TLR4 stimulation, which plays a key role for the activation of NF-κB through the regulation of TAK1 activation (Wi et al., 2014). Moreover, we found that the ubiquitinated ECSIT by TRAF6 specifically interacts with p65/p50 NF-κB components through the ubiquitination chain at the K372 residue of ECSIT, and then the ECSIT/p65/p50 complex leads to the translocation into the nucleus, resulting in the regulation of NF-κB-dependent gene expression (Mi Wi et al., 2015).

Peroxiredoxin-6 (Prdx6) is the prototype and the only mammalian 1-Cys Prdx member (Rhee et al., 2001; Woo et al., 2003). Prdx6 has been widely studied in cells and animal models for its antioxidant properties (Wood et al., 2003; Rhee et al., 2005), which provides protection against the harmful consequences of oxidative stress (Chen et al., 2000). Its expression has been described in several cell compartments including the cytoplasm, secretory organelles, and lysosomes (Immenschuh and Baumgart-Vogt, 2005; Wu et al., 2006; Liu et al., 2012). After ischemia or ischemia/reperfusion (I/R) injury, in which the production of ROS induced by TLR activation is one of the most important factors in the pathogenesis (Jaeschke et al., 1988; Tsung et al., 2005; Evankovich et al., 2010), Prdx6 expression is lost in the cytoplasm and markedly increases in the mitochondria (Eismann et al., 2009). Although the molecular mechanism is as yet largely unexplored, deletion of Prdx 6 exacerbates lipopolysaccharide (LPS)-induced acute lung injury and inflammation with increased oxidative stress, inflammatory responses, and matrix degradation, all of which are partially dependent on the NF-κB, extracellular signal-regulated kinase, and c-Jun N-terminal kinase pathways (Yang et al., 2011).

Here we show that Prdx6 is critically implicated in bactericidal activity and NF-κB activity induced by TLR4. Prdx6 involved these processes through the molecular association with TRAF6. Prdx6-TRAF6 interaction inhibited the association of ECSIT to TRAF6 in the mitochondria and cytoplasm, leading to suppression of mROS generation and NF-κB activation mediated by the TRAF6-ECST complex.

## Materials and methods

### Cell lines and reagents

HEK293T cells (ATCC, Rockville, MD) were cultured in Dulbecco's modified Eagle's medium (DMEM, Gibco, Detroit, MI) supplemented with 10% fetal bovine serum (FBS, Gibco). 293/TLR4 cells (293/TLR4-MD2-CD14, Invivogen, San Diego, CA) were cultured in DMEM supplemented with 10 mg/mL blasticidin and 50 mg/mL HygroGold (Invivogen) as per the manufacturer's instruction. THP-1 human monocytic leukemia cells (ATCC) were maintained in RPMI 1640 supplemented with 10% FBS, penicillin–streptomycin, and β-mercaptoethanol. Lentivirus containing small hairpin RNA (shRNA) targeting human Prdx6 (sc-62896-V) and control shRNA lentivirus (sc-108080) were purchased from Santa Cruz Biotechnology (Santa Cruz, CA). THP-1 cells were cultured in wells of a 24-well plate (2 × 10^5^ cells/well) and infected with control shRNA lentivirus for the generation of control (Ctrl) THP-1 cells and lentivirus containing shRNA targeting human Prdx6 for the generation of Prdx6 knockdown (Prdx6^KD^) THP-1 cells, respectively, according to the manufacturer's protocol. Cells were maintained in puromycin-containing (4–8 μg/ml) medium. MitoTracker Green FM was obtained from Thermo Fisher Scientific (Waltham, MA). The following antibodies and reagents were used: Prdx6 (Abcam, Cambridge, MA), GRIM19 (Abcam), Myc (Cell Signaling Technology, Beverley, MA), Flag, (Cell Signaling Technology), TRAF6 (Cell Signaling Technology), ECSIT (Abcam), 4′,6′-diamindino-2-phenylindole (DAPI; Thermo Fisher Scientific), MitoSOX Red (Molecular Probes, Invitrogen, Carlsbad, CA), CM-H2DCFDA (Invitrogen), and lipopolysaccharide (LPS, L6143; Sigma-Aldrich, St. Louis, MO).

### Cellular fractionation and mitochondrial isolation

293/TLR4 cells were stimulated with or without 500 ng/ml LPS for different times (0, 15, 30, 45, 60 min). Cellular fractionation and mitochondrial isolation protocol was carried out as previously described (West et al., 2011).

### ROS measurements and staining

Control (Ctrl) and Prdx6^KD^ THP-1 cells were treated with or without 500 ng/ml LPS for 60 min. Culture medium was removed, cells were washed with phosphate buffered saline (PBS) then incubated with MitoSOX Red (to measure the mROS superoxide) and/or 5-(and-6)-chloromethyl-2′,7′-dichlorodihydrofluorescein diacetate (CM-H_2_DCFDA, to measure total cellular H_2_O_2_) at 2.5 μM final concentration in serum-free RPMI 1640 for 15 to 30 min at 37°C. Cells were washed with warmed PBS (37°C), removed from plates with cold PBS containing 1 mM EDTA by pipetting, pelleted at 1,500 rpm for 3 min, immediately re-suspended in cold PBS containing 1% FBS, and subjected to fluorescence-activated cell sorting (FACS) analysis using a FACScalibur apparatus (Becton Dickinson, San Diego, CA). All ROS experiments shown are representative of three independent experiments. For the immunofluorescence microscopy, cells were mounted with Prolong Gold anti-fade reagent (Molecular Probes) and imaged on a LSM 710 laser-scanning confocal microscope (Carl Zeiss, Jena, Germany).

### Immunofluorescence confocal microscopy

For all microscopy images, 293/TLR4 cells were grown on coverslips and stimulated with or without 500 ng/ml LPS for 30 min and stained with MitoTracker FM (Invitrogen Ltd, Paisley, UK). After washing, cells were fixed with 4% paraformaldehyde for 20 min, permeabilized with 0.1% Triton X-100 in PBS for 5 min, blocked with PBS containing 10% FBS for 30 min, and stained with primary antibodies (anti-Prdx6). Cells were then stained with secondary antibody, Alexa Fluor® 647 anti-Mouse IgG (H+L) (Jackson ImmunoResearch Laboratories). Cells were stained with 4′,6-diamidino-2-phenylindole (Sigma-Aldrich) and mounted with Prolong Gold anti-fade reagent (Molecular Probes). Cells were imaged on a LSM 710 laser-scanning confocal microscope (Carl Zeiss). The overlap coefficient were calculated using the ZEN 2011 program, which evaluated more than ten cells from three images for each condition.

### Salmonella infection assay

Salmonella infection protocol was previously described (Blanc-Potard and Groisman, 1997; Valle and Guiney, 2005). Briefly, 5 × 10^5^ THP-1 cells were cultured in fresh RPMI 1640 complete medium without antibiotics for 1 day, and treated with 12-*O*-tetradecanoylphorbol-13-acetate (PMA, 20 ng/ml) and seeded into culture wells at a concentration of 7 × 10^5^ cells/ml. The next day, nonadherent cells were removed and replaced with fresh complete medium without antibiotics. The cells were infected with *Salmonella typhimurium* wild type (14028s strain) at a multiplicity of infection of 10 bacteria/cell. Culture plates were centrifuged at 200 × g for 5 min and incubated at 37°C for 30 min to allow phagocytosis to occur. The medium was then replaced with fresh medium containing gentamicin (20 μg/ml) and incubated for different times. The total cell population in the well was harvested. An aliquot of the harvested cell population was centrifuged, the macrophages were lysed by 0.5% deoxycholate in Dulbecco's PBS, and the bacteria were diluted and plated on LB agar. The percentage survival was obtained by dividing the number of bacteria recovered after 6 h or 12 h by the number of bacteria present at time 0 and multiplying by 100. All experiments were done in duplicate on at least three independent occasions.

### Plasmids

The following plasmids were used: Flag-tagged TRAF6, Flag-tagged ECSIT, Myc-tagged ECSIT and Myc-tagged Prdx6, as previously described (Kim et al., 2014; Wi et al., 2014; Mi Wi et al., 2015; Moon et al., 2015). Flag-tagged TRAF6 truncated mutants were generated with specific primers as described in the Supplementary Information.

### Western blotting and immunoprecipitation assay

Western blotting and immunoprecipitation were performed as described previously (Kim et al., 2012, 2014; Yong Kim et al., 2013; Lee et al., 2016). Briefly, HEK293T cells were co-transfected with the designated vectors, as indicated in the Figures. After 38 h, the cells were extracted and immunoprecipitated with anti-Flag or anti-Myc antibody, followed by immune blotting with antibodies to anti-Myc, or anti-Flag. For endogenous immunoprecipitation assay, Ctrl THP-1 and Prdx6^KD^ THP-1 cells were treated with or without LPS (500 ng/ml) for 60 min, respectively. immunoprecipitation assay was performed in lysates with IgG antibody and anti-TRAF6 antibody, and then IB assay was performed with anti-TRAF6, anti-ECSIT, and anti-Prdx6 antibodies.

### Measurement of proinflammatory cytokines and NF-κB DNA-binding assay

Ctrl THP-1 or Prdx6^KD^ THP-1 cells were untreated or treated with LPS (200 ng/ml) for 9 h and the supernatants were harvested. The levels of human IL-1β and IL-6 were measured in the supernatants according to the manufacturer's protocol (R&D Systems, Minneapolis, MN). Ctrl THP-1 or TRAF6^KD^ THP-1 cells were transiently transfected with vector control, Flag-TRAF6, or Myc-Prdx6 using Neon transfection system (Invitrogen). At 36 h post-transfection, the cells were untreated or treated with LPS (200 ng/ml) for 9 h and the supernatants were harvested. The level of human IL-6 was measured in the supernatants according to the manufacturer's protocol (R&D Systems). For p65- or p50-DNA-binding assay, Ctrl THP-1 or Prdx6^KD^ THP-1 cells were treated for 6 h with or without LPS (200 ng/ml), and then nuclear proteins were prepared with the CelLytic NuCLEAR extraction kit in accordance with the manufacturer's protocol (Sigma-Aldrich). Activities of the transcription factors p65 or p50 were determined with the TransAM NF-κB transcription factor assay kit according to the manufacturer's instructions (Active Motif North America, Carlsbad, CA).

### NF-κB-dependent luciferase reporter assay

Ctrl THP-1 and Prdx6^KD^ THP-1 cells were transiently transfected with different vectors including vector control, Myc-Prdx6, Flag-ECSIT, and Flag-TRAF6, as indicated in the Figures, using Neon transfection system (Invitrogen), together with the pBIIx-luc NF-κB-dependent reporter construct and the Renilla luciferase vector (Promega, Madison, WI). At 36 h post-transfection, the cells were untreated or treated with LPS (200 ng/ml) for 6 h and lysed, and luciferase activity was measured using a dual luciferase assay kit (Promega).

### Microarray analysis

Microarray analysis, raw data preparation, and statistical analysis were performed as described previously (Oh et al., 2011; Kim et al., 2012). The protocols are described in detail in the Supplementary Information.

### RNA isolation and qRT–PCR analyses

Control (Ctrl) and Prdx6^KD^ THP-1 cells were untreated or treated with LPS (200 ng/ml) for different times (0, 6, 9 h). Total RNA was using TRIzol method (Invitrogen) and then was reverse-transcripted to single strand cDNA using amfiRivert II cDNA Synthesis Master Mix (GenDEPOT, Barker, TX) according to the manufacturer's instructions. Primers used in this experiment were purchased from Qiagen (Valencia, CA): hIL-1β (PPH00171C-200), hIL-8 (PPH00568A-200), hNFKB1 (PPH00204F-200), hCD44 (PPH00114A-200), hRELB (PPH00287A-200), and hCCL5 (PPH00703B-200). Tag PCR Master Mix (Qiagen) was used according to the manufacturer's protocols to perform RT-PCR reactions on QIAGEN's real-time PCR cycler, the Rotor-Gene Q. The amplification reactions were performed with the following conditions: 5 min at 95°C, 45 cycles of 95°C for 15 s, 55°C for 40 s, 72°C for 30 s. All quantitative measurements were carried out in triplicate and normalized to the internal glyceraldehyde-3-phosphatedehydrogenase (GAPDH) control for every reaction. Results were represented as mean value ± SEM of the mean from triplicate samples.

### Statistical analysis

*In vitro* data are presented as mean ± SEM of the mean from triplicate samples. Statistical differences were analyzed by either ANOVA or Student's *t*-test using GraphPad Prism 5.0 (GraphPad Software, San Diego, California).

## Results

### Prdx6 is a negative regulator of mROS generation and is involved in bactericidal activity

**Figure 1:**
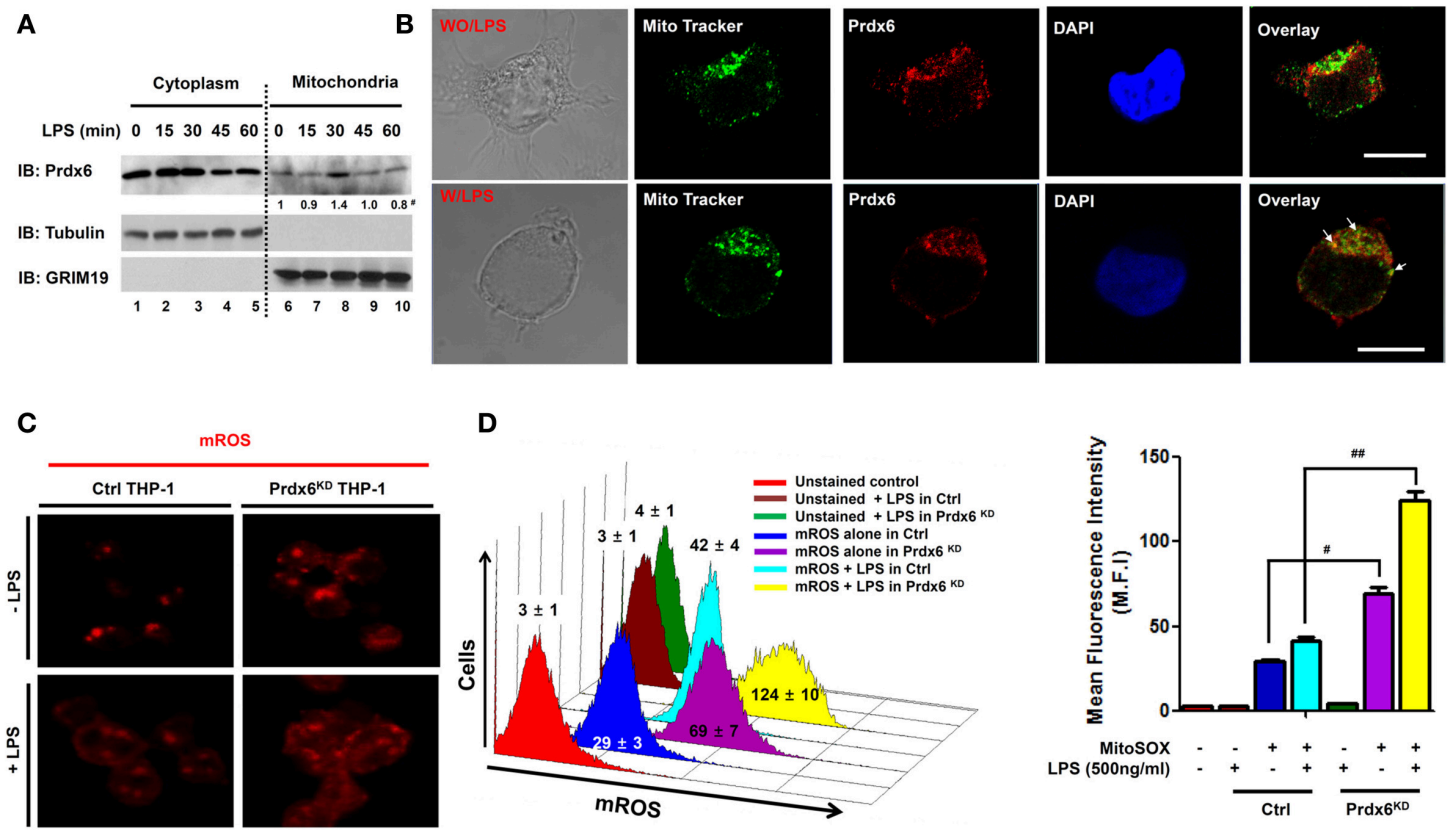
**Peroxiredoxin-6 negatively regulates mROS production in the mitochondria. (A)** 293/TLR4 cells were stimulated with LPS (500 ng/ml) for the indicated times, cells were fractionated and extracts were western-blotted with antibodies for Prdx6, tubulin, or GRIM19. ^#^Densitometry data (normalized to GRIM19). **(B)** 293/TLR4 cells were unstimulated or stimulated with LPS (500 ng/ml) for 30 min, fixed, immunostained with Mito Tracker (green) and anti-Prdx6 (red), and counterstained with 4′, 6-diamono-2-phenylindole (DAPI; blue). Data are representative of three independent replicates; scale bar, 10 μm. **(C)** Ctrl and Prdx6^KD^ THP-1 cells were unstimulated or stimulated with LPS (500 ng/ml) for 60 min, stained with MitoSOX (mitochondria ROS, mROS) and analyzed by immunofluorescence microscopy. Data are representative of three independent replicates. **(D)** Ctrl and Prdx6^KD^ THP-1 cells that were unstimulated or stimulated with LPS (500 ng/ml) for 60 min, stained with MitoSOX and analyzed by FACS. All error bars represent ± SEM of the mean fluorescence intensity (M.F.I) from triplicate samples. ^#^*p* = 0.0008, ^*##*^*p* = 0.0002.

After ischemia or I/R, Prdx6 expression no longer occurs in the cytoplasm and appears in the mitochondria (Eismann et al., 2009). The authors also reported that the increased hepatocellular injury in Prdx6-knockout mice was critically associated with increased mitochondrial generation of hydrogen peroxide (H_2_O_2_), implicating the mitochondrial trafficking of Prdx6 with mROS generation. Additionally, it has been reported that the generation of mROS is regulated by TLR stimulation and is involved in bactericidal activity (West et al., 2011; Geng et al., 2015). Based on these previous results, we examined whether the mitochondrial trafficking of Prdx6 could be also regulated by TLR stimulation and be involved in mROS generation. 293/TLR4 cells were not stimulated or were stimulated with LPS, a TLR4 agonist, for different times, and the cytoplasm and mitochondria fractions were isolated. Immunoblotting predominantly revealed Prdx6 in the cytoplasm with marginal detection in the mitochondria in the absence of LPS (Figure 1A, lanes 1 and 6). In the presence of LPS stimulation the mitochondrial content of Prdx6 was significantly increased (Figure 1A, lane 8). Consistent with this, confocal microscopy revealed mitochondrial trafficking of Prdx6. Upon LPS stimulation, Prdx6 was significantly co-localized with MitoTracker Green FM (Figure 1B, without LPS vs. with LPS in overlay), suggesting that the mitochondrial trafficking of Prdx6 is induced by TLR4 stimulation.

To correlate the mitochondrial trafficking of Prdx6 induced by TLR4 with mROS generation, we generated control (Ctrl) THP-1 cells and Prdx6-knockdown (Prdx6^KD^) THP-1 cells using control shRNA lentivirus and Prdx6 shRNA-containing lentiviral particles, respectively (Figure S1). Consistent with a previous report (West et al., 2011), the mROS level was significantly increased by TLR4 stimulation (Figures 1C,D, without LPS vs. with LPS in Ctrl THP-1). We found that the mROS level was significantly higher in Prdx6^KD^ THP-1 cells than in Ctrl THP-1 cells in the absence of TLR4 stimulation (Figures 1C,D, Ctrl vs. Prdx6^KD^ THP-1 cells without LPS). According to LPS stimulation, interestingly, the levels were markedly elevated in the Prdx6^KD^ THP-1 cells, as compared to Ctrl THP-1 cells (Figures 1C,D, Ctrl vs. Prdx6^KD^ THP-1 cells with LPS). Moreover, the levels of cellular H_2_O_2_ were also significantly higher in Prdx6^KD^ THP-1 cells than in Ctrl THP-1 in the absence or presence of LPS stimulation (Figure S2), supporting the functional role of Prdx6 as a cellular anti-oxidant enzyme capable of neutralizing cellular H_2_O_2_. These results suggest that Prdx6 is translocated into the mitochondria by TLR4 stimulation and is negatively implicated in the production of mROS in the mitochondria.

**Figure 2:**
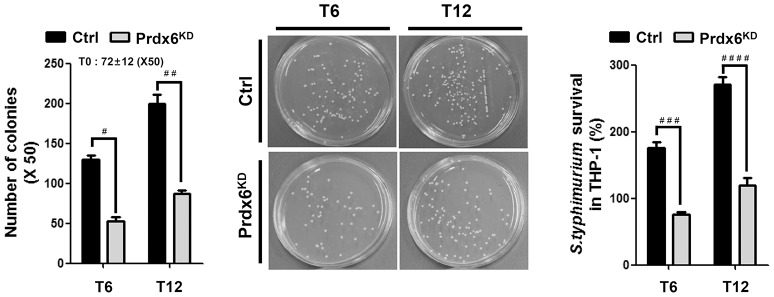
**Salmonella survival assay in Prdx6-knockdown THP-1 cells**. Control (Ctrl) and Prdx6-knockdown (Prdx6^KD^) THP-1 cells were infected with *Salmonella* wild type (14028s strain) at a multiplicity of infection of 10 bacteria/cell as described in Methods. Cells were lysed by 0.5% deoxycholate in Dulbecco's PBS, and the bacteria were diluted (x 50) and plated on LB agar. The number of colonies was counted and represented. The number of colonies at T = 0 was represented as an average of both cell lines, controls and Prdx6^KD^ cells. The percentage survival was obtained by dividing the number of bacteria recovered after 6 h (T6) or 12 h (T12) by the number of bacteria present at time 0 (T0) and multiplying by 100. All error bars represent ± SEM of three independent experiments. ^#^*p* = 0.00013, ^*##*^*p* = 0.00037, ^*###*^*p* = 0.00003, ^*####*^*p* = 0.00004.

mROS plays an important for the macrophage bactericidal activity (Murphy, 2009; Koopman et al., 2010; West et al., 2011; Geng et al., 2015). Having shown that Prdx6^KD^ THP-1 cells had increased mROS (Figures 1C,D), we examined the functional effect of Prdx6 in bactericidal activity. Ctrl or Prdx6^KD^ THP-1 cells were infected with the gram-negative, facultative intracellular pathogen *Salmonella typhimurium*, and survival of the bacterium was measured. The number of colonies at T = 0 was similar in both controls and Prdx6^KD^ cells, as represented in Figure 2, suggesting that the Prdx6-knockdown did not affect the initial invasion and uptake of *S. typhimurium*. Interestingly, the number of colonies was significantly increased in Ctrl THP-1 cells in a time-dependent manner, whereas a significant decrease was evident in Prdx6^KD^ THP-1 cells (Figure 2, Ctrl vs. Prdx6^KD^). In addition, the survival of *S. typhimurium* was significantly attenuated in Prdx6^KD^ THP-1 cells (Figure 2, Ctrl vs. Prdx6^KD^). Together these results suggest that Prdx6 as a negative regulator of mROS may be implicated in bactericidal activity, although the molecular mechanism by which Prdx6 is involved in the production of mROS induced by TLR4 stimulation is needed to be provided.

### Prdx6 interacts with TRAF6 and leads to mitochondrial localization

**Figure 3:**
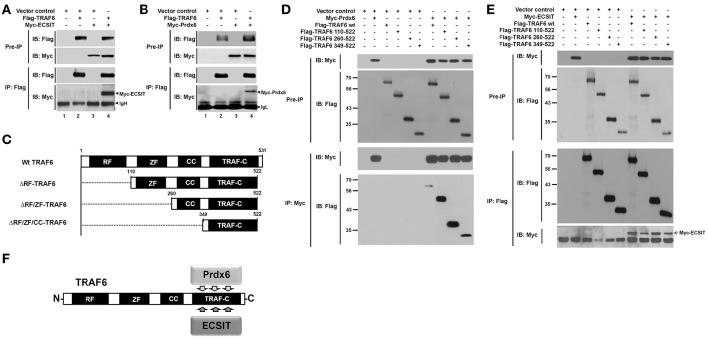
**Prdx6 interacts with the TRAF-C domain of TRAF6. (A)** HEK293T cells were transiently transfected with vector control, Flag-TRAF6, or Myc-ECSIT, as indicated. After 38 h, immunoprecipitation (IP) assay with anti-Flag antibody was performed, followed by immune blotting (IB) with antibodies to anti-Myc or anti-Flag. **(B)** HEK293T cells were transiently transfected with vector control, Flag-TRAF6, or Myc-Prdx6, as indicated. After 38 h, IP assay with anti-Flag antibody was performed, followed by IB with antibodies to anti-Myc or anti-Flag. **(C)** Schematic representation of TRAF6 truncated mutants used in this study. Each truncated mutant was generated, as described in Materials and Methods. **(D)** HEK293T cells were transfected with vector control, Myc-Prdx6, Flag-TRAF6 wild type (wt), Flag-TRAF6 110-522, Flag-TRAF6 260-522, or Flag-TRAF6 349-522, as indicated. At 38 h after transfection, transfected cells were extracted, immunoprecipitated with anti-Myc antibody, and then an IB assay was performed with anti-Flag or anti-Myc antibody. **(E)** HEK293T cells were vector control transfected or transfected with Myc-ECSIT, Flag-TRAF6 wt, Flag-TRAF6 110-522, Flag-TRAF6 260-522, or Flag-TRAF6 349-522, as indicated. At 38 h after transfection, transfected cells were extracted, immunoprecipitated with anti-Flag antibody, and then an IB assay was performed with anti-Flag or anti-Myc antibody. **(F)** A schematic model of the molecular interaction between TRAF6 and Prdx6 or ECSIT.

Having shown the effects of Prdx6 on mROS generation and bactericidal activity, we next explored the molecular mechanism by which Prdx6 is negatively involved in the production of mROS. A previous report has shown that the engagement of TLRs leads to the translocation of mitochondria to phagosomes, mediated by the assembly of a complex of the ubiquitin ligase TRAF6 and the mitochondrial complex I–assembly factor ECSIT, which results in augmentation of mROS production and bactericidal activity (West et al., 2011). Therefore, we raised a possibility that Prdx6 is involved in the assembly of TRAF6-ECSIT complex. To examine the possibility, Flag-TRAF6 was transiently expressed into HEK293T cells along with Myc-ECSIT or Myc-Prdx6, and then an immunoprecipitation assay was performed with anti-Flag antibody. Myc-ECSIT or Myc-Prdx6 was significantly co-precipitated with Flag-TRAF6 (Figures 3A,B, respectively), whereas no significant interaction between Myc-Prdx6 and Flag-ECSIT could be seen (Figure S3). To confirm whether Prdx6 and TRAF6 proteins are translocated into the mitochondria in response to LPS stimulation, 293/TLR4 cells were stimulated with or without LPS, and the cellular localizations of Prdx6 and TRAF6 into the mitochondria were assessed by confocal microscopy. Consistent with a previous report (West et al., 2011), the localization of TRAF6 into mitochondria was increased in the presence of LPS stimulation, as compared to that in the absence of LPS stimulation (Figures S4A,B, Mito/TRAF6). The overlap coefficient to quantify the co-localization was significant (Figures S4A,B, Mito/TRAF6: 0.48 ± 0.04 in without LPS vs. 0.77 ± 0.07 in with LPS). Consistent with Figure 1B, Prdx6 was significantly co-localized with mitochondria marker in the presence of LPS stimulation (Figures S4A,B, Mito/Prdx6: 0.38 ± 0.08 in without LPS vs. 0.68 ± 0.05 in with LPS). Based on these results, we supposed that, if Prdx6 is involved in the mROS production through the assembly of TRAF6-ECSIT complex in mitochondria (West et al., 2011), it might be related with the TRAF6 protein.

### Prdx6 interrupts the interaction between TRAF6 and ECSIT

**Figure 4:**
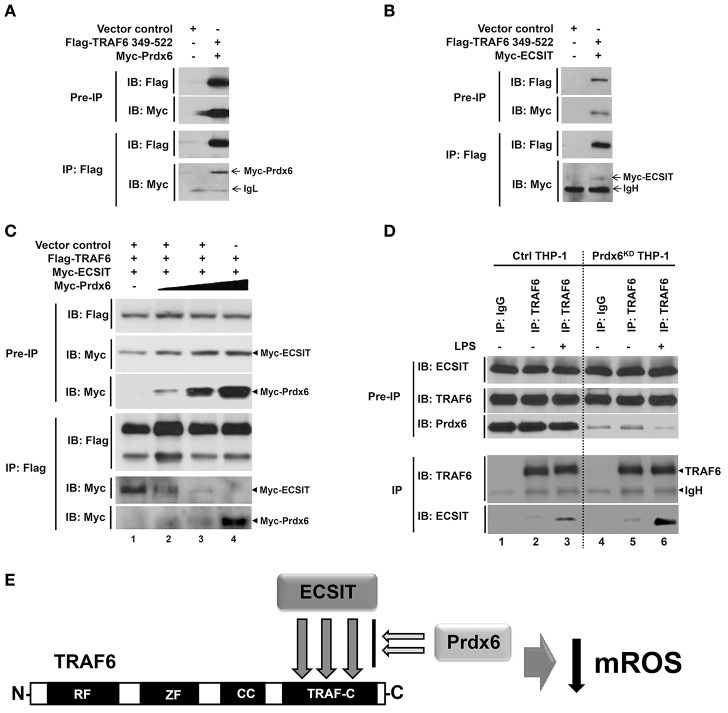
**Prdx6 interrupts the TRAF6-ECSIT interaction**. **(A)** HEK293T cells were transfected with vector control, Flag-TRAF6 349-522, or Myc-Prdx6, as indicated. At 38 h after transfection, transfected cells were extracted, immunoprecipitated with anti-Flag antibody, and then an IB assay was performed with anti-Flag or anti-Myc antibody. **(B)** HEK293T cells were transfected with vector control, Flag-TRAF6 349-522, or Myc-ECSIT, as indicated. At 38 h after transfection, transfected cells were extracted, immunoprecipitated with anti-Flag antibody, and then an IB assay was performed with anti-Flag or anti-Myc antibody. **(C)** HEK293T cells were transfected with vector control, Flag-TRAF6, Myc-ECSIT, or different concentrations of Myc-Prdx6, as indicated. At 38 h after transfection, transfected cells were extracted, immunoprecipitated with anti-Flag antibody, and then an IB assay was performed with anti-Flag or anti-Myc antibody. **(D)** Ctrl THP-1 and Prdx6^KD^ THP-1 cells were treated with or without LPS (500 ng/ml) for 60 min, respectively. IP assay was performed in lysates with IgG antibody as control and anti-TRAF6 antibody, and then IB assay was performed with anti-TRAF6, anti-ECSIT, and anti-Prdx6 antibodies. **(E)** Model of how Prdx6 inhibits the production of mROS through the inhibition of the interaction between TRAF6 and ECSIT.

To investigate whether Prdx6 is affected on the formation of TRAF6-ECSIT complex, we first determine the interaction site of TRAF6 to ECSIT or Prdx6. In order to do that, TRAF6 truncated mutants were generated (Figure 3C), as described in Materials and Methods. Flag-TRAF6 wild type (wt) and Flag-tagged truncated mutants of TRAF6 were transfected with Myc-Prdx6 or Myc-ECSIT, and then immunoprecipitation assay was performed with anti-Myc or anti-Flag antibody. Interestingly, Myc-Prdx6 efficiently precipitated Flag-TRAF6 wt and all truncated mutants of TRAF6 (Figure 3D). Moreover, Flag-TRAF6 wt and all truncated mutants were specifically precipitated with Myc-ECSIT (Figure 3E). These results indicate that Prdx6 or ECSIT interacts with the C-terminal TRAF-C domain of TRAF6 (Figure 3F). To confirm the specific interaction, Myc-Prdx6 or Myc-ECSIT was transfected into HEK293T cells along with Flag-TRAF6 349-522 containing the TRAF-C domain of TRAF6, and then immunoprecipitated using an anti-Flag antibody. Consistently, Flag-TRAF6 349-522 was specifically precipitated with Myc-Prdx6 (Figure 4A) or Myc-ECSIT (Figure 4B).

Next, we examined whether Prdx6 and ECSIT are competitively interacted with the TRAF-C domain of TRAF6. Flag-TRAF6 and Myc-ECSIT were transiently transfected into HEK293T cells with different concentrations of Myc-Prdx6, and then immunoprecipitated using an anti-Flag antibody. As expected, Flag-TRAF6 strongly precipitated Myc-ECSIT in the absence of Myc-Prdx6 (Figure 4C, lane 2). Interestingly, the interactions between Flag-TRAF6 and Myc-ECSIT were greatly attenuated in the presence of Myc-Prdx6, whereas significant dose-dependent increases of the interaction between Flag-TRAF6 and Myc-Prdx6 could be observed (Figure 4C, lanes 2–5), strongly suggesting that Prdx6 and ECSIT are competitively interacted with the TRAF-C domain of TRAF6. To verify the effect of Prdx6 on ECSIT-TRAF6 interaction upon LPS stimulation, endogenous IP assay was performed in Ctrl THP-1 and Prdx6^KD^ THP-1 cells in the presence or absence of LPS stimulation. Ctrl THP-1 and Prdx6^KD^ THP-1 cells were treated with or without LPS, and then immunoprecipitation assay was performed with anti-TRAF6 antibody. Consistent with previous reports, endogenous ECSIT protein was significantly pull-downed with endogenous TRAF6 in the presence of LPS stimulation (Figure 4D, lane 3). Interestingly, the endogenous interaction between ECSIT and TRAF6 were significantly increased in the Prdx6^KD^ THP-1 cells, as compared with that of Ctrl THP-1 cells (Figure 4D, lane 3 vs. lane 6). As depicted in Figure 4E, collectively, these results suggest that Prdx6 interacts with TRAF6 via its TRAF-C domain, and the Prdx6-TRAF6 complex is translocated into the mitochondria. The Prdx6-TRAF6 complex inhibits the association of ECSIT to TRAF6 in the mitochondria, resulting in the inhibition of mROS production.

### Prdx6 is negatively involved in NF-κB activation induced by TLR4 stimulation

**Figure 5:**
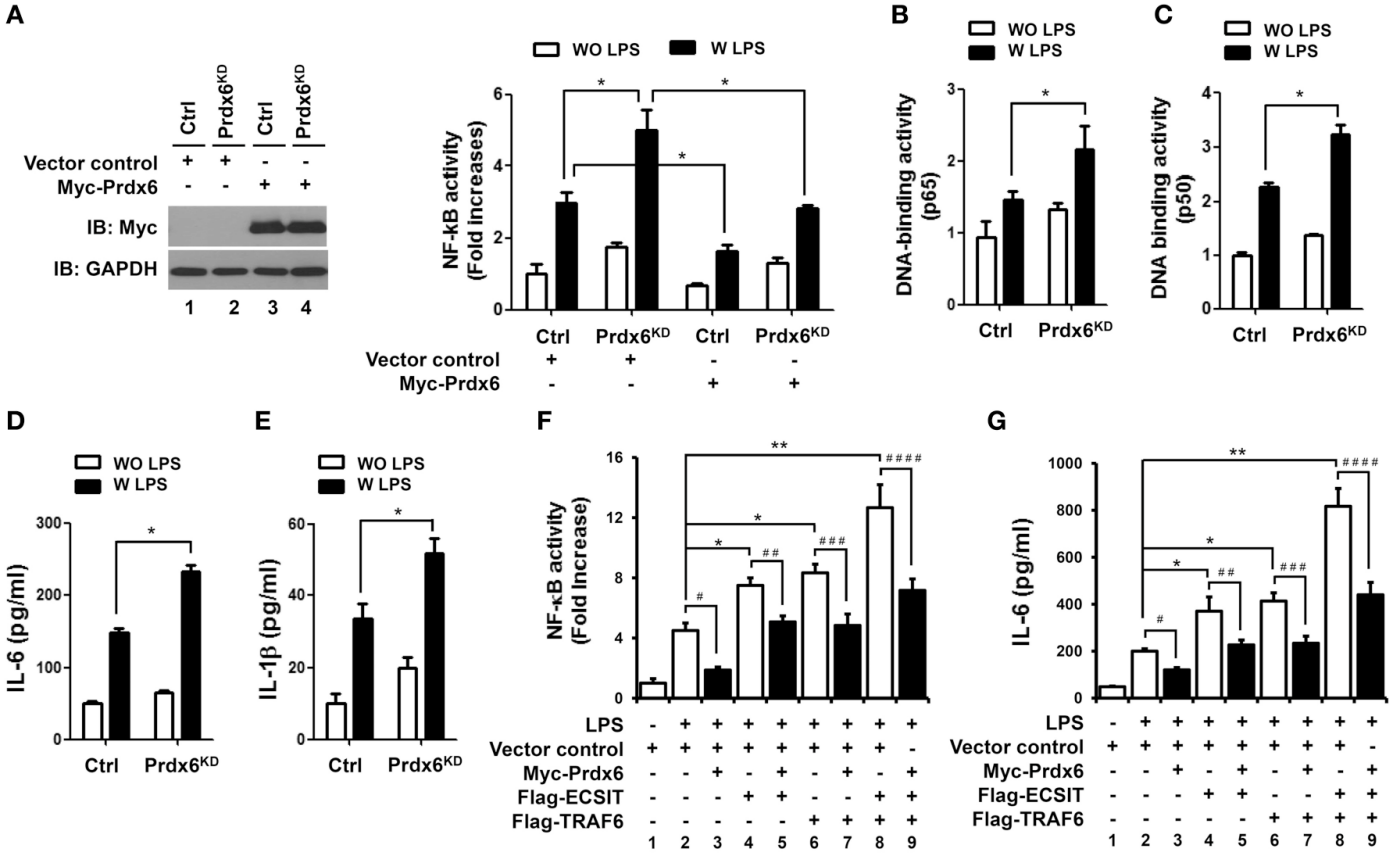
**Prdx6-knockdown THP-1 cells display enhanced NF-κB activity induced by TLR4 stimulation. (A)** Ctrl and Prdx6^KD^ cells were transfected with a vector control or Myc-Prdx6 vector together with pBIIx-luc and Renilla luciferase vector. Thirty six hours after transfection, the Myc-Prdx6 expression was confirmed by Western blotting (left). Cells were untreated or treated with LPS (200 ng/ml) for 6 h and then analyzed for luciferase activity. Results are expressed as the fold-induction in luciferase activity relative to that in untreated cells. All error bars represent ± SEM of the mean from triplicate samples. ^*^p < 0.05. **(B,C)** Ctrl and Prdx6^KD^ cells were untreated or treated with LPS (200 ng/ml) for 6 h and then analyzed for p65- or p50-DNA binding activity using the manufacturer's protocol. All error bars represent ± SEM of the mean from triplicate samples. ^*^p < 0.05. **(D,E)** Ctrl and Prdx6^KD^ cells were untreated or treated with LPS (200 ng/ml) for 9 h, and production of IL-6 **(D)** and IL-1β **(E)** were analyzed by enzyme-linked immunosorbent assay (ELISA). All error bars represent ± SEM of the mean from triplicate samples. ^*^p < 0.05. **(F)** THP-1 cells were transfected with vector control, Flag-TRAF6, Flag-ECSIT, or Myc-Prdx6 vector, as indicated, together with pBIIx-luc and Renilla luciferase vector. Thirty six hours after transfection, cells were untreated or treated with LPS (200 ng/ml) for 6 h and then analyzed for luciferase activity. Results are expressed as the fold-induction in luciferase activity relative to that in untreated cells. All error bars represent ± SEM of the mean from triplicate samples. ^*^p < 0.05 and ^**^p < 0.01, ^#^*p* = 0.0006, ^*##*^*p* = 0.0013, ^*###*^*p* = 0.0097, ^*####*^*p* = 0.0189. **(G)** THP-1 cells were transfected with vector control, Flag-TRAF6, Flag-ECSIT, or Myc-Prdx6 vector, as indicated. Thirty six hours after transfection, cells were untreated or treated with LPS (200 ng/ml) for 9 h, and production of IL-6 was analyzed by enzyme-linked immunosorbent assay (ELISA). All error bars represent ± SEM of the mean from triplicate samples. ^*^p < 0.05 and ^**^p < 0.01, *p* = 0.0002, ^*##*^*p* = 0.0119, ^*###*^*p* = 0.0012, ^*####*^*p* = 0.0042.

Beyond the role of TRAF6-ECSIT in mitochondria, it has been reported that the complex also plays a pivotal role for the activation of NF-κB in Toll/IL-1 signal transduction pathways (Kopp et al., 1999). Interestingly, a previous report has shown that the deletion of peroxiredoxin-6 exacerbates lipopolysaccharide-induced inflammation with increased oxidative stress and inflammatory responses (Yang et al., 2011). Based on the above results that Prdx6 interrupts the molecular interaction between TRAF6 and ECSIT, we supposed that the negative regulation of Prdx6 in TLR4 signaling might be implicated in the inhibition of TRAF6-ECSIT formation in the cytoplasm. As expected, overexpression of Prdx6 in 293/TLR4 cells significantly led to suppressions of NF-κB activity and p65-DNA binding activity induced by TLR4 stimulation (Figures S5A,B). To confirm the functional roles of Prdx6 in TLR4 signaling for the activation of NF-κB, Ctrl and Prdx6^KD^ THP-1 cells were stimulated with or without LPS, and NF-κB activities were measured using a luciferase assay. Upon LPS stimulation, NF-κB activity was markedly higher in Prdx6^KD^ THP-1 cells than in Ctrl THP-1 cells, whereas the activity was significantly attenuated by the expression of Prdx6 in both Ctrl THP-1 and Prdx6^KD^ THP-1 cells (Figure 5A). Furthermore, p65- or p50-DNA binding activity was also enhanced in Prdx6^KD^ THP-1 cells compared with Ctrl THP-1 cells (Figure 5B, p65; Figure 5C, p50). Consistently, the production of the proinflammatory cytokines IL-6 and IL-1β was significantly higher in Prdx6^KD^ THP-1 cells treated with LPS than in Ctrl THP-1 cells (Figure 5D, IL-6; Figure 5E, IL-1β). To further verify the functional effect of Prdx6, THP-1 cells were transiently transfected with vector control, Flag-ECSIT, Flag-TRAF6, or Flag-ECSIT and Flag-TRAF6 in the presence or absence of Myc-Prdx6, and then NF-κB activity and IL-6 production were measured in response to TLR4 stimulation. The overexpression of ECSIT, TRAF6, or ECSIT and TRAF6 enhanced NF-κB reporter activity and IL-6 production, as compared to vector control transfectant (Figures 5F,G, column 2 vs. column 4, column 6, and column 8). In contrast, these activities were markedly attenuated by the overexpression of Prdx6 (Figures 5F,G: column 4 vs. column 5, column 6 vs. column 7, and column 8 vs. column 9), suggesting Prdx6 is negatively involved in the TLR4-induced NF-κB activation.

**Figure 6:**
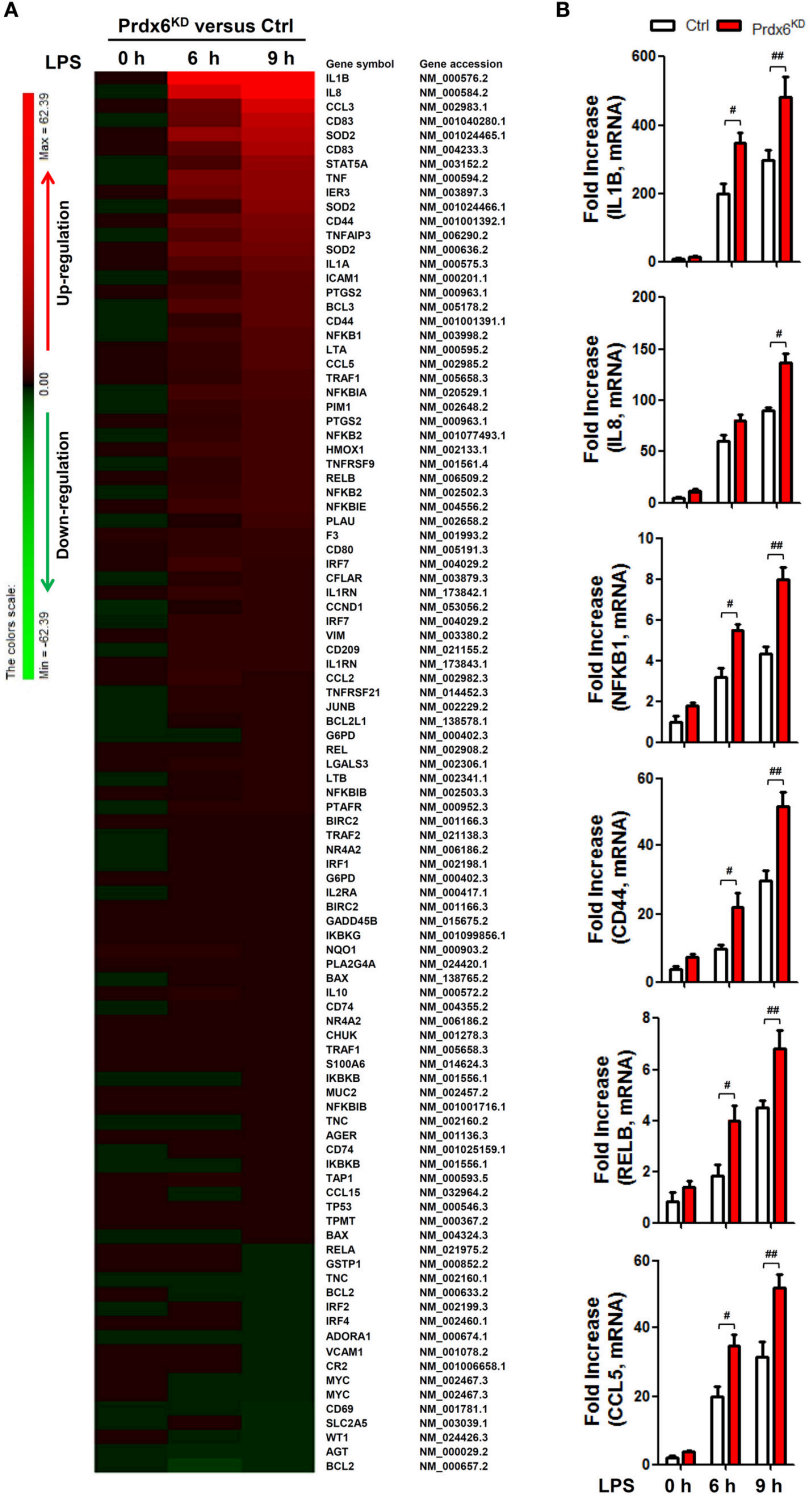
**NF-κB-dependent gene expressions induced by TLR4 are increased in Prdx6^**KD**^ THP-1 cells. (A)** Comparison of NF-κB-dependent gene expression in control (Ctrl) vs. Prdx6^KD^ THP-1 cells. Ctrl and Prdx6^KD^ THP-1 cells were untreated or treated with LPS (200 ng/ml) for different times, as indicated. Total RNA was extracted, and microarray analysis was performed, as described in Methods. NF-κB-dependent gene expressions were compared in Ctrl THP-1 vs. Prdx6^KD^ THP-1 cells. Gene expression levels are indicated with color bars (Red, up-regulated genes; green, down-regulated genes) **(B)** Ctrl and Prdx6^KD^ THP-1 cells were untreated or treated with LPS (200 ng/ml) for different times, as indicated. Total RNA was extracted, and quantitative RT-PCR analysis with specific primers to IL-1β, IL-8, NFκB1, CD44, RELB, and CCL5, was performed. All error bars represent ± SEM of the mean from triplicate samples. IL-1β:^#^*p* = 0.0014 and ^*##*^*p* = 0.0179; IL-8: ^#^*p* = 0.0015; NFκB1:^#^*p* = 0.0034 and ^*##*^*p* = 0.0004; CD44: ^#^*p* = 0.0222 and ^*##*^*p* = 0.0063; RELB: ^#^*p* = 0.0072 and ^*##*^*p* = 0.0102: CCL5; ^#^*p* = 0.0078 and ^*##*^*p* = 0.0112.

Next, we confirmed whether Prdx6 affects expression of NF-κB-dependent genes by TLR4 stimulation. Ctrl THP-1 and Prdx6^KD^ THP-1 cells were untreated or treated with LPS for different times (Figure 6A), and microarray analysis was performed to evaluate gene expression. Upon LPS stimulation, NF-κB-dependent gene expressions sorted out from microarray data were markedly elevated in Prdx6^KD^ THP-1 cells, as compared with those of Ctrl THP-1 cells (Table S1; Figure 6A, Prdx6^KD^ vs. Ctrl THP-1 cells). To verify their expression, we performed quantitative real-time PCR analysis with specific primers targeted to IL-1β, IL-8, NFκB1, CD44, RELB, and CCL5 genes. As expected, these genes were up-regulated in Ctrl THP-1 cells treated with LPS in a time-dependent manner (Figure 6B, open bars). In Prdx6^KD^ THP-1 cells, interestingly, these genes were significantly elevated, as compared with those of Ctrl THP-1 cells (Figure 6B, open bars in Ctrl vs. closed red bars in Prdx6^KD^).

## Discussion

The phagocytic response in the innate immune system involves the production of ROS via the phagosomal NADPH-oxidase-dependent respiratory burst. This is a necessary effector response for the destruction of intracellular microbes (Underhill and Ozinsky, 2002; Lambeth, 2004). In addition to NADPH-oxidase, the mitochondrial oxidative phosphorylation machinery generates ROS when electrons prematurely escape oxidative phosphorylation complexes I and III, and react with molecular oxygen to generate superoxide (Murphy, 2009; Koopman et al., 2010). The regulatory mechanisms by which mROS is produced by innate immune signaling could be essential for antibacterial responses, with signal transmission from phagosomes acting to recruit mitochondria for effective phagosome-mitochondrion function and bactericidal activity (West et al., 2011; Geng et al., 2015). TLR-mediated signaling induces the generation of mROS by the translocation of mitochondria to phagosomes, which is mediated by the assembly of a complex of the ubiquitin ligase TRAF6 and the mitochondrial complex I–assembly factor ECSIT (West et al., 2011). Interestingly, a recent study has shown that Mst1 and Mst2 kinases are critically involved in the optimal ROS production and bactericidal activity by increasing the activation of the small GTPase Rac and mitochondrial trafficking and the mitochondrion-phagosome association through the assembly of a TRAF6-ECSIT complex (Geng et al., 2015). These studies strongly suggest a pivotal role of the assembly of TRAF6-ECSIT complex induced by TLR stimulation for the production of mROS and bactericidal activity. In this study, we have demonstrated that Prdx6 negatively regulates the generation of mROS. Prdx6 is localized into the mitochondria in response to LPS stimulation. Biochemical study revealed that Prdx6 wild type and C47A Prdx6 catalytic mutant were interacted with TRAF6 in similar levels (Figure S6, lanes 2 vs. 3 in IB: Myc), supposing that the enzymatic mutant of Prdx6 does not affect on the molecular interaction between Prdx6 and TRAF6. Moreover, the Prdx6-TRAF6 interaction inhibited the association of TRAF6-ECSIT complex. Interestingly, we found that the mitochondrial localization of Prdx6 was significantly attenuated in the TRAF6-knockdown THP-1 cells in response to LPS stimulation, as compared with that of control THP-1 cells (Figure S7, lanes 6 vs. 8 in IB: Prdx6), indicating that the localization of Prdx6 might be accompanied with TRAF6. Furthermore, we found that the mROS level was significantly higher in Prdx6^KD^ THP-1 cells than in Ctrl THP-1 cells, and the *Salmonella* survival rate was markedly attenuated in Prdx6^KD^ THP-1 cells. These results suggest that Prdx6 might negatively regulate the generation of mROS through the interruption of TRAF6-ECSIT complex, and involve bactericidal activity.

Besides the functional role of TRAF6-ECSIT complex in the mitochondria, TRAF6 and ECSIT proteins have been critically implicated in the Toll/IL-1 signal transduction pathways (Kopp et al., 1999; Wi et al., 2014; Mi Wi et al., 2015). TRAF6 is a member of the TRAF family with E3 ubiquitin ligase activity. It is crucial in activating IκB kinase (IKK) and mitogen-activated protein kinase, leading to activation of NF-κB (Akira et al., 2006). ECSIT was first discovered as an adaptor protein involved in coupling TLR and TRAF6 to MEKK-1 and NF-κB (Kopp et al., 1999). In addition, our recent studies revealed new regulatory roles for the activation of NF-κB in response to TLR4 stimulation (Wi et al., 2014; Mi Wi et al., 2015). ECSIT forms the signaling complex, including TAK1 and TRAF6, and the complex is pivotal in TLR4-mediated signals to activate NF-κB (Wi et al., 2014). ECSIT also specifically interacts with p65/p50 NF-κB proteins through the ubiquitination of the ECSIT lysine 372 residue, and is then translocated into the nucleus resulting in the increase of proinflammatory cytokines and the enhancement of NF-κB-dependent gene expression in response to TLR4 stimulation (Mi Wi et al., 2015). Interestingly, we found that the overexpression of Prdx6 led to the inhibition of NF-κB induced by TLR4, whereas Prdx6^KD^ THP-1 cells displayed enhanced production of pro-inflammatory cytokines including interleukin-6 and -1β, and the up-regulation of NF-κB-dependent genes induced by TLR4 stimulation. Having shown that Prdx6 interacted with TRAF6, and the Prdx6-TRAF6 interaction inhibited the association of ECSIT protein to TRAF6, we speculate that Prdx6 might be negatively involved in the TLR4-induced NF-κB activation through the inhibition of interaction between TRAF6 and ECSIT.

**Figure 7:**
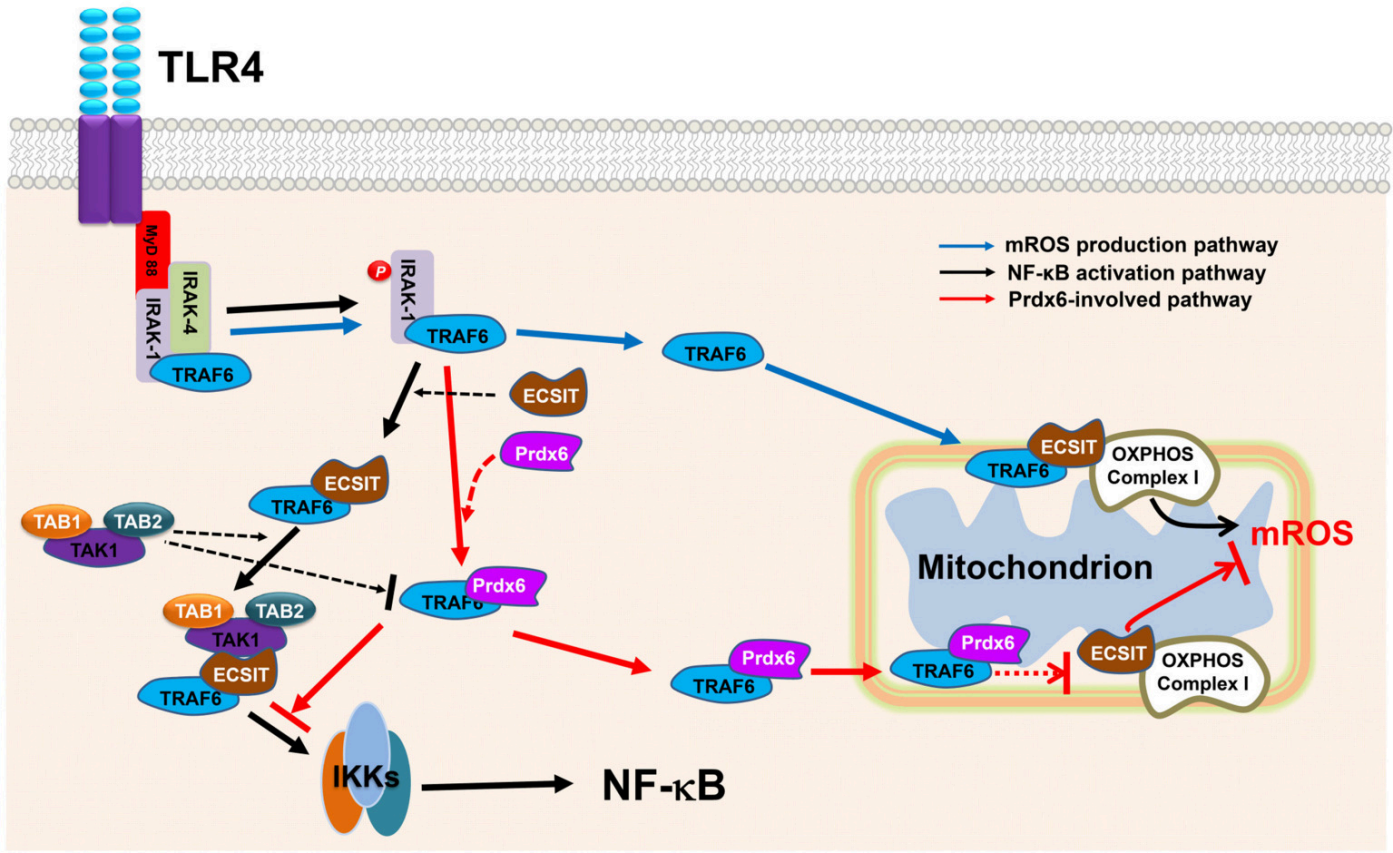
**Model detailing the negative role of Prdx6 in mROS generation and NF-κB activation induced by TLR4**. On TLR4 stimulation, MyD88 binds to the cytoplasmic portion of the TLRs through interactions between individual TIR domains. IRAKs, such as IRAK-4 and IRAK- 1, and TRAF6 are recruited to the receptor, and then phosphorylated IRAK-1 (by IRAK-4) dissociates from the receptor together with TRAF6. TRAF6 translocates into the mitochondria and associates with ECSIT-OXPHOS complex I, leading to the production of mROS (blue arrow line). In the meantime, the TRAF6 further associates with TAB1–TAK1–TAB2 complex through the cytoplasmic ECSIT protein and activates IKKs complex, eventually leading to activations of NF-κB (black arrow line). We speculate that Prdx6 interacts with TRAF6, which leads to two inhibitory effects (red arrow line); one is to inhibit the association of TRAF6-ECSIT- OXPHOS complex I in the mitochondria for mROS production, another is to inhibit the interaction between TRAF6 and ECSIT and thereby leads to the inhibition of associations of TAB1–TAK1–TAB2 complex to TRAF6-ECSIT for the activation of IKKs.

The present findings demonstrate that Prdx6 inhibits the association of TRAF6-ECSIT complex in mitochondria and cytoplasm. The interruption of assembly of TRAF6-ECSIT complex is critical in the suppression of mROS generation in mitochondria and the inhibition of NF-κB activation in cytoplasm induced by TLR4, respectively. Having shown essential roles of TRAF6-ECSIT complex exerting mROS regulation and NF-κB activation, we propose a plausible model of the negative regulation of Prdx6 in these pathways. As depicted in Figure 7, upon TLR4 stimulation MyD88 binds to the cytoplasmic portion of the TLRs through interactions between individual TIR domains. IRAKs including IRAK-4 and IRAK-1, and TRAF6 are recruited to the receptor. IRAK-1 is phosphorylated by IRAK-4 and dissociates from the receptor together with TRAF6. TRAF6 further interacts with TAK1, TAB1, and TAB2 leading to the activation of IKKs. Simultaneously, TRAF6 translocates into the mitochondria, where mROS is produced by the OXPHOS complex I associated with the TRAF6-ECSIT proteins, leading to the bactericidal activity. In the meantime, Prdx6 interacts with TRAF6, and that translocates into the mitochondria. The TRAF6-Prdx6 complex does not associate with the ECSIT- OXPHOS complex I because Prdx6 masks the interaction domain of TRAF6 to ECIST, resulting in the suppression of mROS production. Additionally, the interaction of Prdx6 to TRAF6 in the cytoplasm inhibits the formation of TRAF6-ECSIT complex, which has been critically implicated in the activation of NF-κB induced by TLR stimulation (Kopp et al., 1999; Wi et al., 2014; Mi Wi et al., 2015), resulting in the inhibition of NF-κB activation.

Inflammatory signals including TLRs need to be tightly controlled in the host because excessive or prolonged inflammatory responses to microbial infection can lead to harmful effects to the host (Beutler, 2004). Varied mechanisms and cellular proteins capable of interrupting TLRs-mediated signaling have been proposed and reported (Trompouki et al., 2003; Boone et al., 2004; Liew et al., 2005; Zhang et al., 2013; Jiao et al., 2015). In line with these findings, our data may contribute to the understanding of cellular and molecular regulations in limiting inflammatory responses, thereby advancing the development of therapeutic targets capable of regulating inflammatory responses.

## Author contributions

EC and KL conceived the study and drafted the paper; YM and SW performed most of the experiments described in the manuscript; DS helped with the experiments. All authors read and approved the manuscript.

### Conflict of interest statement

The authors declare that the research was conducted in the absence of any commercial or financial relationships that could be construed as a potential conflict of interest.
