# Supplementary material for: Peroxiredoxin-6 Negatively Regulates Bactericidal Activity and NF-κB Activity by Interrupting TRAF6-ECSIT Complex
Source: Front Cell Infect Microbiol. 2017 Mar 24;7:94. doi: 10.3389/fcimb.2017.00094 (PMC5364181; doi:10.3389/fcimb.2017.00094)
Supplement: Supplementary file 2 [file Image1.pdf]

Peroxiredoxin 6 negatively regulates bactericidal activity and NF-κB activity by interrupting TRAF6-ECSIT complex

Yoon Min[1§], Sae Mi Wi[1§], Dongwoo Shin[1], Eunyoung Chun[2*] and Ki-Young Lee[1*]

[1]Department of Molecular Cell Biology and Samsung Biomedical Research Institute, Sungkyunkwan University School of Medicine, Suwon 440-746, Republic of Korea,

[2]Department of Immunology and Infectious Diseases, Harvard School of Public Health, and the Department of Medicine, Harvard Medical School, Boston, MA 02115, USA

Running title: Negative Regulation of Peroxiredoxin 6

Keywords: Peroxiredoxin 6, mROS, Bactericidal activity, NF-κB, TRAF6, ECSIT

Corresponding author: Eunyoung Chun (echun@hsph.harvard.edu) and Ki-Young Lee (thylee@skku.edu)

§Yoon Min and Sae Mi Wi contributed equally to this paper

Supplementary methods

Generation of Prdx6-knockdown THP-1 cells.

THP-1 human monocytic leukemia cells (ATCC) were maintained in RPMI 1640 supplemented with 10% FBS, penicillin–streptomycin, and β-mercaptoethanol. Lentivirus containing small hairpin RNA (shRNA) targeting human Prdx6 (sc-62896-V) and control shRNA lentivirus (sc-108080) were purchased from Santa Cruz Biotechnology (Santa Cruz, CA). THP-1 cells were cultured in wells of a 24-well plate (2×10^5 cells/well) and infected with lentivirus according to the manufacturer's protocol. Control (Ctrl) and Prdx6 knockdown ($Prdx6^{KD}$) THP-1 cells were generated and maintained in puromycin-containing (4 to 8 μg/ml) medium. To evaluate the knockdown efficacy, western blotting analysis was performed with anti-Prdx6 or anti-GAPDH antibody.

ROS measurements and staining

Control (Ctrl) and $Prdx6^{KD}$ THP-1 cells were treated with or without 500 ng/ml LPS for 60 min. Culture medium was removed, cells were washed with phosphate buffered saline (PBS) then incubated with 5-(and-6)-chloromethyl-2′,7′-dichlorodihydrofluorescein diacetate (CM-H_2DCFDA, to measure total cellular H_2O_2) at 2.5 μM final concentration in serum-free RPMI 1640 for 15 to 30 min at 37 °C. Cells were washed with warmed PBS (37 °C), removed from plates with cold PBS containing 1 mM EDTA by pipetting, pelleted at 1,500 rpm for 3 min, immediately re-suspended in cold PBS containing 1% FBS, and subjected to fluorescence-activated cell sorting (FACS) analysis using a FACScalibur apparatus (Becton Dickinson, San Diego, CA). All ROS experiments shown are representative of three independent experiments. For the immunofluorescence microscopy, cells were mounted with Prolong Gold anti-fade reagent (Molecular Probes) and imaged on a Zeiss LSM 710 laser-scanning confocal microscope (Carl Zeiss, Jena, Germany).

Salmonella infection assay

5×10^5 THP-1 cells were cultured in fresh RPMI 1640 complete medium without antibiotics and infected with *Salmonella typhimurium* wild type (14028s strain) at a multiplicity of infection of 10 bacteria/cell. Culture plates were centrifuged at 200 × g for 5 min and incubated at 37°C for 30 min to allow phagocytosis to occur. The medium was then replaced with fresh medium containing gentamicin (20 μg/ml) and incubated for different times. The total cell population in the well was harvested. An aliquot of the harvested cell

population was centrifuged, the macrophages were lysed by 0.5% deoxycholate in Dulbecco's PBS, and the bacteria were diluted and plated on LB agar. The percentage survival was obtained by dividing the number of bacteria recovered after 6 h or 12 h by the number of bacteria present at time 0 and multiplying by 100. All experiments were done in duplicate on at least three independent occasions.

Generation of TRAF6 truncated mutants

Flag-tagged TRAF6 truncated mutants were generated with specific primers: Flag-tagged 110-522, forward 5'-GCGAAGCTTATGGAA ATACTGCTGGAAAATCAACT-3' and reverse 5'-AACTCGAGCTATACCCCTGCATCAGTACT-3'; Flag-tagged 260-522, forward 5'- ATAAGCTTATGCGCCACCTACAAGAGAACA-3' and reverse 5'-AACTCGAGCTATACCCCTGCATCAGTACT-3'; Flag-tagged 349-522 TRAF6, forward 5'-GCGCAAGCTTATGTGCAATGGAATTTATATTTGGAAG-3' and reverse 5'-AACTCGAGCTATACCCCTGCATCAGTACT-3'.

Immunoprecipitation assay

HEK293T cells were co-transfected with mock, Myc-Prdx6, or Flag-ECSIT, as indicated in the supplemental Fig. S3. After 38 h, the cells were extracted and immunoprecipitated with anti-Myc antibody, followed by immune blotting with antibodies to anti-Myc, or anti-Flag.

Immunofluorescence confocal microscopy

For all microscopy images, 293/TLR4 cells were grown on coverslips and stimulated with or without 500 ng/ml LPS for 30 min and stained with Mito Tracker Orange CMTMRos (Invitrogen). After washing, cells were fixed with 4% paraformaldehyde for 20 min, permeabilized with 0.1% Triton X-100 in PBS for 5 min, blocked with PBS containing 10% FBS for 30 min, and stained with primary antibodies (anti-Prdx6 or anti-TRAF6). Cells were then stained with secondary antibodies: Alexa Fluor® 488 anti-goat IgG (H+L) (Jackson ImmunoResearch Laboratories, West Grove, PA), Alexa Fluor® 647 anti-Mouse IgG(H+L) (Jackson ImmunoResearch Laboratories), and Alexa Fluor® 568 Anti-Rabbit IgG 568nm (Invitrogen). Cells were stained with 4′,6-diamidino-2-phenylindole (Sigma-Aldrich) and

mounted with Prolong Gold anti-fade reagent (Molecular Probes). Cells were imaged on a LSM 710 laser-scanning confocal microscope (Carl Zeiss). The overlap coefficient were calculated using the ZEN 2011 program, which evaluated more than ten cells from three images for each condition

NF-κB-dependent luciferase reporter assay

293/TLR4 cells were transfected with a mock or Myc-Prdx6 vector, using Neon transfection system (Invitrogen), together with the pBIIx-luc NF-κB-dependent reporter construct and the Renilla luciferase vector (Promega, Madison, WI). At 36 h post-transfection, the cells were untreated or treated with LPS (200 ng/ml) for 6 h and lysed, and luciferase activity was measured using a dual luciferase assay kit (Promega).

p65- DNA-binding assay

293/TLR4 cells were transfected with a mock or Myc-Prdx6 vector. 38 h after transfection, cells were untreated or treated with LPS (200 ng/ml) for 6 h, and then nuclear proteins were prepared with the CelLytic NuCLEAR extraction kit in accordance with the manufacturer's protocol (Sigma-Aldrich). Activities of the transcription factors p65 were determined with the TransAM NF-κB transcription factor assay kit according to the manufacturer's instructions (Active Motif North America, Carlsbad, CA).

Microarray analysis

Control (Ctrl) and Prdx6^{KD} THP-1 cells were untreated or treated with LPS (200 ng/ml) for different times. Total RNA was isolated, and the RNA purity and integrity were evaluated using a ND-1000 Spectrophotometer (NanoDrop Technologies, Wilmington, DE) and model2100 Bioanalyzer (Agilent Technologies, Palo Alto, CA). Total RNA was amplified and purified using TargetAmp-Nano Labeling Kit for Illumina Expression BeadChip (EPICENTRE, Madison, WI) to yield biotinylated cRNA according to the manufacturer’s instructions. For the hybridization, 750 ng of labeled cRNA samples were hybridized to each Human HT-12 v4.0 Expression Beadchip for 17 h at 58°C, according to the manufacturer's instructions (Illumina, Inc., San Diego, CA). Detection of array signal was carried out using fluorolink streptavidin-Cy3 (GE Healthcare Bio-Sciences, Little Chalfont, UK) following the bead array manual. For the raw data preparation and statistic analysis, the quality of

hybridization and overall chip performance were monitored by visual inspection of both internal quality control checks and the raw scanned data. Raw data were extracted using the software provided by the manufacturer (Illumina GenomeStudio v2011.1; Gene Expression Module v1.9.0). Statistical significance of the expression data was determined using fold change. For a DEG set, hierarchical cluster analysis was performed using complete linkage and Euclidean distance as a measure of similarity. Gene-Enrichment and Functional Annotation analysis for significant probe list was performed using DAVID (http://david.abcc.ncifcrf.gov/home.jsp). All data analysis and visualization of differentially expressed genes was conducted using R 3.0.2 (www.r-project.org).

Supplementary figure legends

FIGURE S1. Generation of Prdx6-knockdown THP-1 cells. THP-1 cells were infected with lentivirus containing shRNA targeted human Prdx6 or control lentivirus according to the manufacture's protocol. Control THP-1 (Ctrl) and Prdx6-knockdown THP-1 ($Prdx6^{KD}$ THP-1) were cultured in puromycin-containing medium (4 μg/ml) for 2 weeks to select stable clones, and immunoblotting with antibody to anti-Prdx6 or anti-GAPDH was performed to evaluate the knockdown efficacy

FIGURE S2. Increase of cellular H_2O_2 in $Prdx6^{KD}$ THP-1 cells. (A) Control (Ctrl) and $Prdx6^{KD}$ THP-1 cells were stimulated with or without 500ng/ml LPS for 60 min, stained with CM-H2DCFDA (cellular H_2O_2) and analysed by immunofluorescence microscopy. Data are representative of three independent replicates. **(B)** Ctrl and $Prdx6^{KD}$ THP-1 cells treated with or without 500ng/ml LPS for 60 min were stained with CM-H_2DCFDA and analysed by FACS. All error bars represent ± SEM of the mean fluorescence intensity (M.F.I) from triplicate samples. * $p < 0.05$, ** $p < 0.01$

FIGURE S3. Prdx6 does not interact with ECSIT. HEK293T cells were co-transfected with mock, Myc-Prdx6, or Flag-ECSIT, as indicated. After 38 h, the cells were extracted and immunoprecipitated with anti-Myc antibody, followed by immune blotting with antibodies to anti-Myc, or anti-Flag.

FIGURE S4. Co-localization of mitochondria-TRAF6 or mitochondria-Prdx6. (A and B) 293/TLR4 cells were not stimulated **(A)** or stimulated **(B)** with 500 ng/ml LPS for 30 min, fixed, immunostained with antibodies for Mito Tracker, TRAF6, and Prdx6, as described in Supplemental methods, and counterstained with DAPI. The overlap coefficient were calculated using the ZEN 2011 program, which evaluated more than ten cells from three images for each condition, and represented as ± SEM.

FIGURE S5. Overexpression of Prdx6 induces suppression of NF-κB activity induced by TLR4. (A) 293/TLR4 cells cells were transfected with a mock or Myc-Prdx6 vector together with pBIIx-luc and Renilla luciferase vector. Twenty four hours after transfection, cells were untreated or treated with LPS (200 ng/ml) for 6 h and then analyzed for luciferase activity. Results are expressed as the fold-induction in luciferase activity relative to that in

untreated cells. All error bars represent ± SEM of the mean from triplicate samples. **(B)** 293/TLR4 cells were transfected with a mock or Myc-Prdx6 vector. 38 h after transfection, cells were untreated or treated with LPS (200 ng/ml) for 6 h and then analyzed for p65-DNA binding activity using the manufacturer's protocol. All error bars represent ± SEM of the mean from triplicate samples. * $p < 0.05$, ** $p < 0.01$.

FIGURE S6. The molecular interaction between Prdx6 wild type or C47A Prdx6 and TRAF6 . HEK293T cells were transiently transfected with vector control, Myc-Prdx6 wild type, Myc-Prdx6 C47A mutant, or Flag-TRAF6, as indicated. After 38 h, immunoprecipitation (IP) assay with anti-Flag antibody was performed, followed by immune blotting (IB) with antibodies to anti-Myc or anti-Flag.

FIGURE S7. The localization of Prdx6 into the mitochondria is attenuated in the $TRAF6^{KD}$ THP-1 cells . Control (Ctrl) THP-1 and TRAF6-knockdown ($TRAF6^{KD}$) THP-1 cells were treated with or without LPS (200 ng/ml) for 30 min. Cells were fractionated and extracts were western-blotted with antibodies for TRAF6, Prdx6, tubulin, or GRIM19. Densitometry of the protein signals was performed by using ImageJ software. $^{\#}$: Densitometry data (normalized to GRIM19 in Ctrl THP-1 and $TRAF6^{KD}$ THP-1 cells)

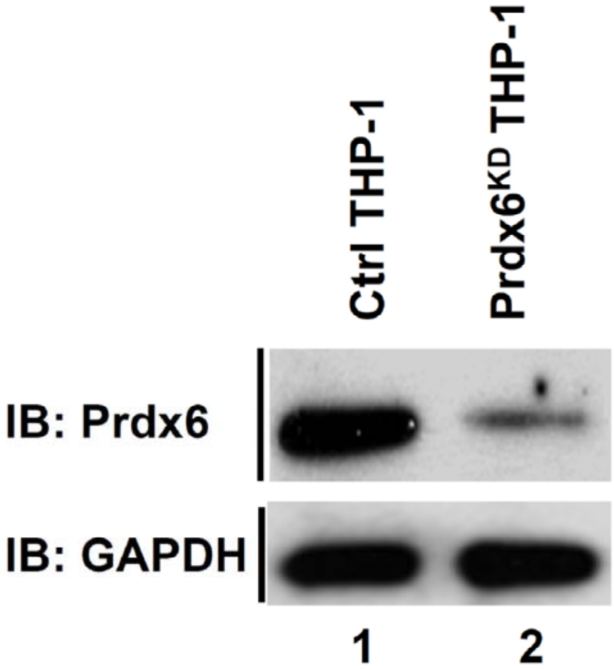

Supplementary Figure S1

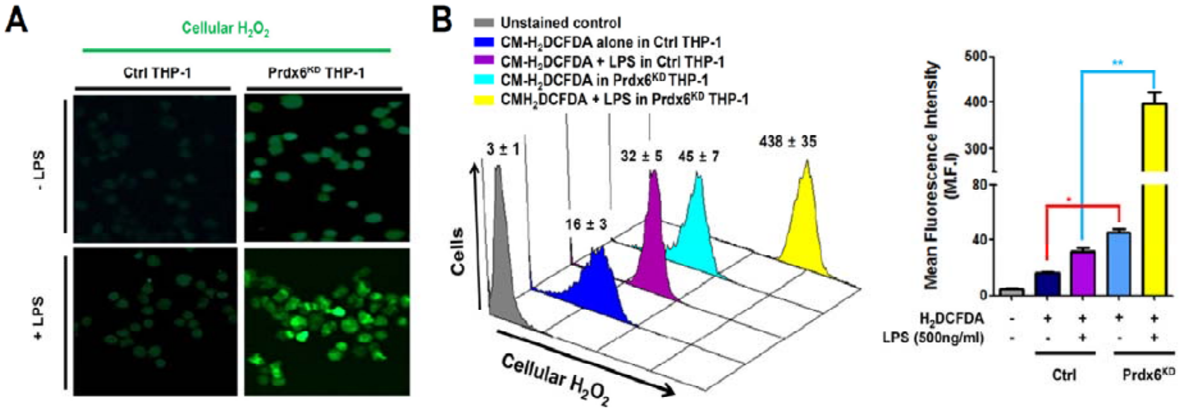

Supplementary Figure S2

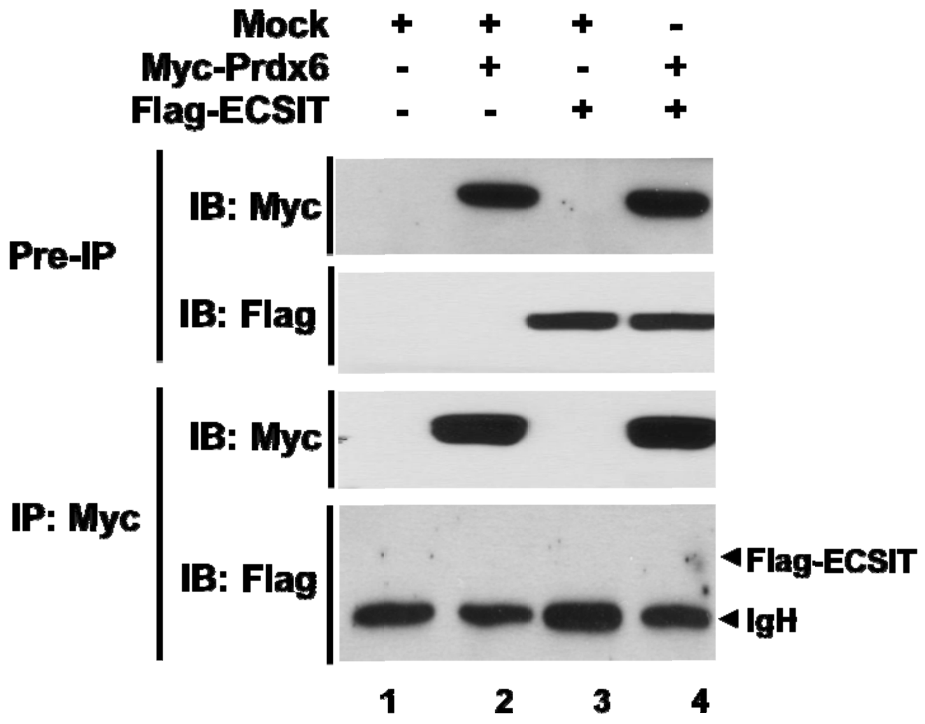

Supplementary Figure S3

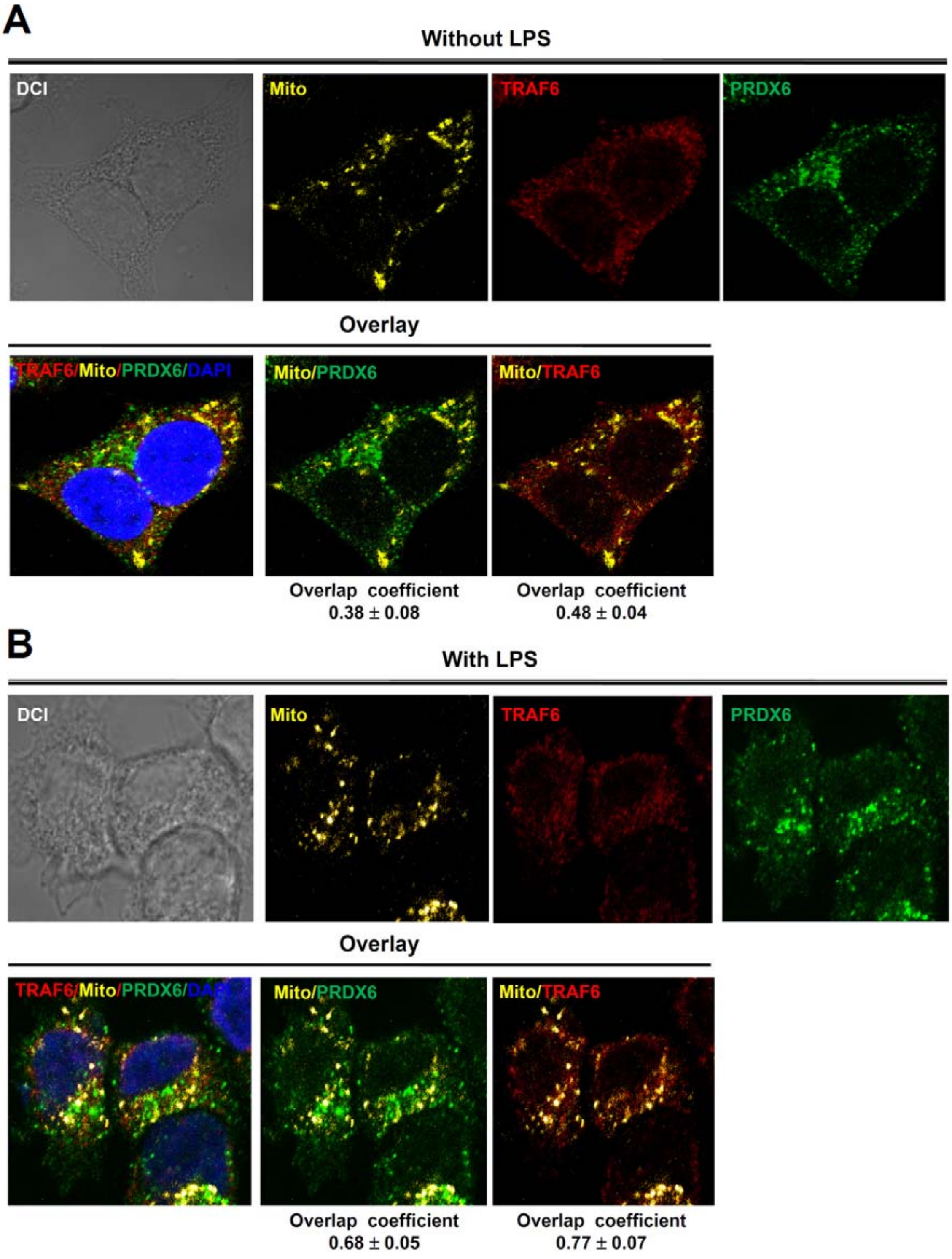

Supplementary Figure S4

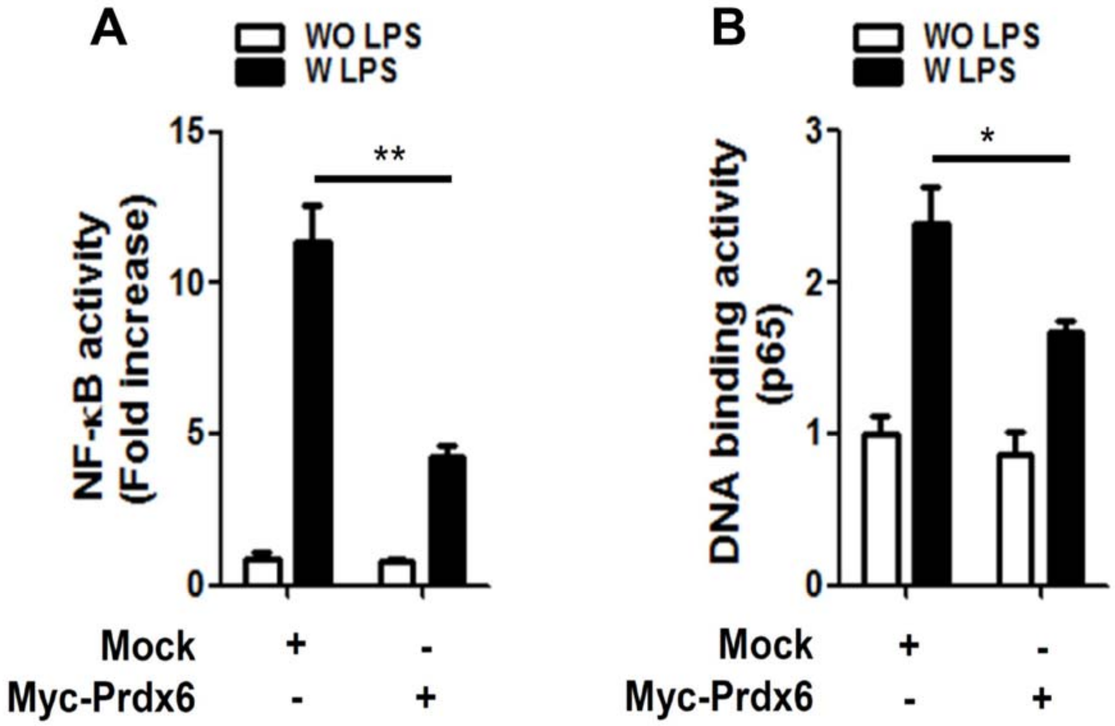

Supplementary Figure S5

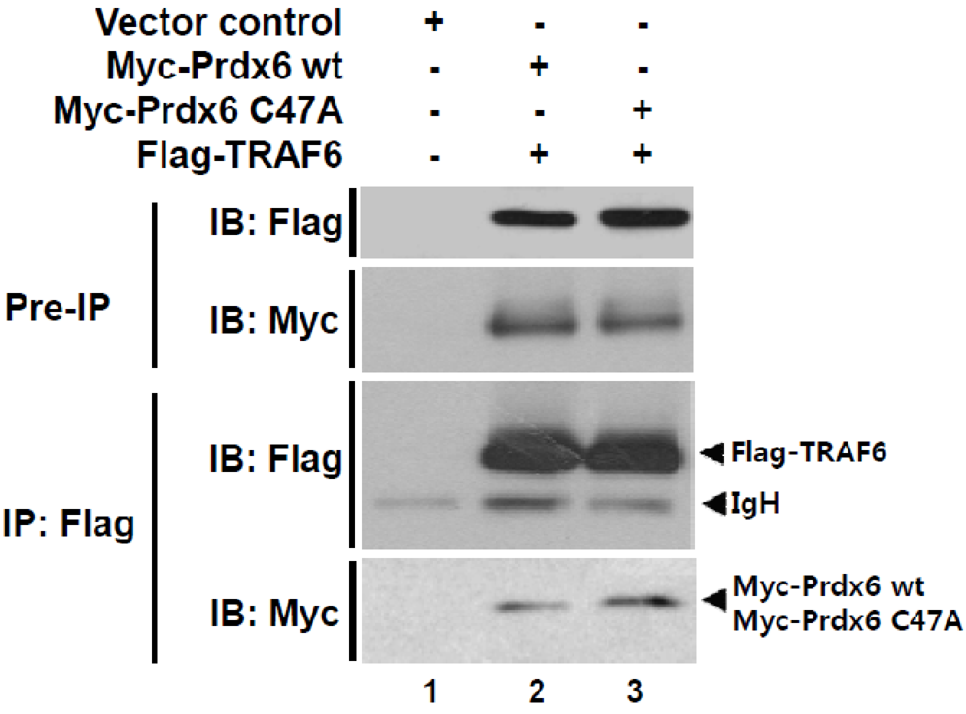

Supplementary Figure S6

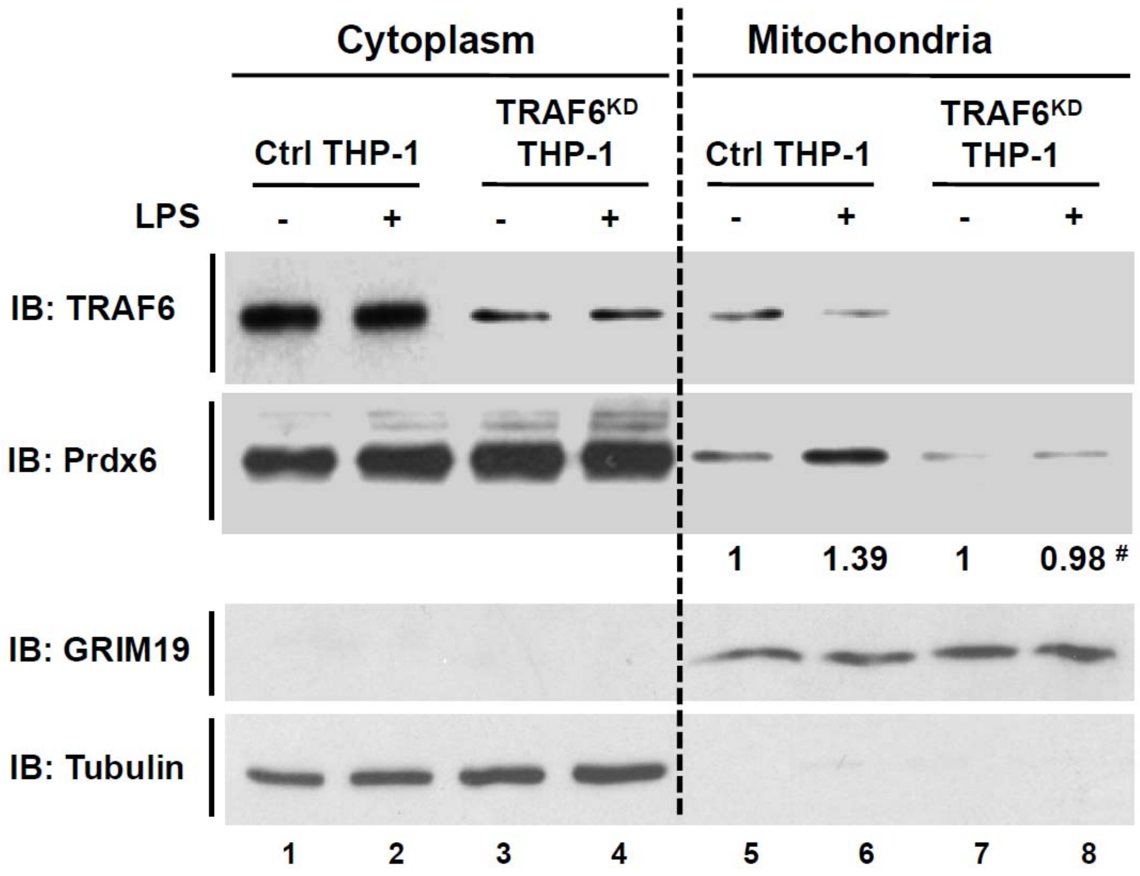

Supplementary Figure S7
